# Association of Weekly Moderate-to-Vigorous Physical Activity with Subjective Vitality and Mental Toughness in Spanish Adolescents Aged 10–16 Years

**DOI:** 10.3390/bs16091660

**Published:** 2026-09-16

**Authors:** Pablo Ramírez-Espejo, José Luis Solas-Martínez, María del Carmen Carcelén-Fraile, Manuel J. de la Torre-Cruz

**Affiliations:** 1 Department of Didactic of Musical, Plastic, and Corporal Expression, University of Jaén, 23071 Jaén, Spain; pramirez@ujaen.es; 2 Department of Education and Sport, Loyola Andalucía University, 14004 Córdoba, Spain; 3 Department of Educational Sciences, Faculty of Social Sciences, Universidad del Atlántico Medio, 35017 Las Palmas de Gran Canaria, Spain; carmen.carcelen@pdi.atlanticomedio.es; 4 Department of Psychology, University of Jaén, 23071 Jaén, Spain; majecruz@ujaen.es

**Keywords:** adolescents, exercise, mental health, vitality, well-being

## Abstract

Physical activity levels decline notably during adolescence, raising concerns due to its impact on present and future psychological health. Understanding how weekly MVPA is associated with psychological functioning during adolescence may provide relevant information for research and health promotion. Therefore, the aim of this study was to examine the association between weekly moderate-to-vigorous physical activity (MVPA) and two key psychological outcomes, subjective vitality and mental toughness, in Spanish adolescents aged 10–16 years, controlling for sex, age, and body mass index (BMI). This cross-sectional study included 286 adolescents (52.4% girls; mean age = 13.34 ± 1.61 years) who participated in the study. MVPA was measured using the PACE+ Adolescent Physical Activity Measure, subjective vitality using the Subjective Vitality Scale, and mental toughness using the Mental Toughness Index. Linear regression and ANCOVA analyses were performed to assess associations. Analyses revealed that higher weekly MVPA was significantly associated with greater subjective vitality and mental toughness, independent of covariates. Active adolescents demonstrated substantially higher subjective vitality (+12.7%) and mental toughness (+10.0%) compared with inactive peers (both *p* < 0.05). Regular engagement in MVPA appears to be associated with higher psychological well-being and resilience during adolescence. These findings support the importance of expanding opportunities for adolescents to engage in MVPA as part of strategies aimed at promoting adaptive psychological capacities.

## 1. Introduction

Preadolescence and adolescence are critical stages of human development, characterized by substantial physical, emotional, and social changes. Lifestyle behaviors established during these formative years have profound implications for current and future health outcomes (Espinoza et al., 2022). Among these behaviors, physical activity (PA), particularly moderate-to-vigorous physical activity (MVPA), has emerged as an important behavioral factor associated with health and well-being (Marquez et al., 2020). According to the World Health Organization, children and adolescents aged 5 to 17 should engage in at least 60 min of MVPA daily (WHO, 2024). Despite these guidelines, global surveillance data indicate a widespread insufficiency: over 80% of adolescents and approximately 31% of adults fail to meet minimum PA recommendations (WHO, 2024). This prevalence of inactivity stems from a complex interplay of factors, including shifts in transportation patterns, marked by a decline in active commuting (Aubert et al., 2022), reduced motivation linked to social difficulties (Sampasa-Kanyinga et al., 2020), the proliferation of recreational screen time (Ramírez-Espejo et al., 2025b), and high academic demands that often displace regular exercise (Wunsch et al., 2017). Current evidence suggests that adolescents who do not engage in regular PA exhibit higher fatigue levels, poorer emotional regulation, lower academic performance, and an increased risk of anxiety and depression (Costa et al., 2021; Hanif et al., 2024; Santos et al., 2023; Solas-Martínez et al., 2025).

In addition to its established relevance for physical health, regular MVPA during adolescence has been associated with several aspects of psychological well-being, including mood, perceived energy, self-esteem, and broader indicators of mental health (Hanif et al., 2024). Participation in physically active and sport-related contexts may also provide opportunities for social interaction, self-regulation, goal pursuit, and coping with challenges. From a physiological perspective, MVPA contributes to cardiovascular fitness, muscular and metabolic health, and healthy physical development (Santos et al., 2023; Solas-Martínez et al., 2025). These multidimensional benefits provide a broader rationale for examining whether weekly MVPA is also associated with subjective vitality and mental toughness during adolescence (Fengjun et al., 2022).

Given the deleterious effects of physical inactivity on psychological well-being, identifying personal resources that can be enhanced through systematic MVPA is crucial. Two constructs emerge as particularly relevant for fostering positive mental health during adolescence: subjective vitality and mental toughness (Fengjun et al., 2022; Santana-Monagas et al., 2023). Subjective vitality represents the conscious experience of possessing energy, enthusiasm, and aliveness (Ryan & Deci, 2008). This construct is considered a central indicator of eudaimonic well-being, consistently correlating with happiness, self-realization, perceived health, and intrinsic motivation (Santana-Monagas et al., 2023; Vergara-Torres et al., 2020). In adolescents, vitality is positively related to a sense of purpose and overall psychological development (Qi et al., 2023). Furthermore, it is linked to internal coping mechanisms that facilitate stress management (Bostic et al., 2000; Castillo et al., 2017) and fosters more satisfying interpersonal relationships (Akin et al., 2016), underscoring its role in socio-emotional adjustment within the school context.

Complementarily, mental toughness constitutes a psychological capacity to effectively navigate challenges, manage pressure, and sustain effort despite adversity (Valadez et al., 2024). This multidimensional construct encompasses emotional control, perseverance, confidence, and commitment (Zalewska et al., 2019). High mental toughness entails robust self-confidence, emotional stability, and the ability to maintain focus in demanding environments (Fengjun et al., 2022). During adolescence, this resource may be relevant to tolerating frustration and overcoming academic and personal obstacles. It serves as a buffer against anxiety, emotional exhaustion, and amotivation, translating into improved school adjustment and enhanced psychological well-being (Brand et al., 2014; Madigan & Nicholls, 2017; Schaefer et al., 2016).

Although subjective vitality and mental toughness are conceptually distinct, examining them together may provide a broader perspective on adolescent psychological functioning. Subjective vitality primarily reflects the perceived availability of energy, aliveness, and psychological activation, whereas mental toughness encompasses psychological resources related to coping with pressure, perseverance, emotional regulation, and persistence in challenging situations (Santana-Monagas et al., 2023; Valadez et al., 2024). Thus, these constructs capture complementary aspects of well-being: one primarily concerns how energized and psychologically alive adolescents feel, while the other concerns how they perceive their capacity to respond to difficulties and sustain adaptive functioning. Considering both outcomes may therefore help clarify whether MVPA is associated with a broader range of psychological resources rather than with a single dimension of well-being (Fengjun et al., 2022; Santana-Monagas et al., 2023).

Recent scholarship underscores the relevant role of PA in promoting psychological well-being during youth (Belaire et al., 2024; Trajković et al., 2023). Among modifiable lifestyle factors, MVPA has been identified as a relevant behavioral factor associated with both subjective vitality and mental toughness (Brand et al., 2017; Molina-García et al., 2011). However, important gaps remain regarding specific psychological resources such as subjective vitality and mental toughness, particularly among Spanish adolescents. Evidence suggests that these variables are critical at this developmental stage, contributing to emotional adjustment, academic motivation, and resilience in the face of school-related and social stressors (Fu et al., 2025; Lu et al., 2025; Ruiz-Ranz & Asín-Izquierdo, 2024). Likewise, MVPA is linked to a heightened sense of competence and improved effort regulation, reinforcing core psychological skills (Brusseau et al., 2018; Csabai et al., 2024). However, research specifically examining these associations in Spanish youth remains limited, and few studies account for differences based on weekly MVPA frequency. Consequently, a deeper understanding of how regular engagement in MVPA is associated with these psychological constructs is required. Thus, the aim of this study was to analyze the association of weekly MVPA with subjective vitality and mental toughness in Spanish students aged 10 to 16 years, controlling for sex, age, and BMI. Based on previous evidence linking physical activity with psychological well-being, vitality, and psychological resources during adolescence, we hypothesized that higher weekly MVPA would be positively associated with subjective vitality (H1) and mental toughness (H2). We also expected adolescents in the higher-frequency MVPA group to show higher levels of subjective vitality and mental toughness than those in the lower-frequency MVPA group.

## 2. Materials and Methods

### 2.1. Participants

A total of 286 Spanish adolescents (52.4% female and 47.6% male) from five secondary schools in Andalusia, southern Spain, took part in this cross-sectional study. The participants were between 10 and 16 years old (13.34 ± 1.61 years), with an average body mass index (BMI) of 21.02 ± 4.53 kg/m^2^. Eligibility criteria included enrollment in one of the participating schools and an age between 10 and 16 years. No additional exclusion criteria based on chronic disease, psychiatric diagnosis, or physical disability were applied unless these conditions prevented completion of the study procedures. Statistically significant differences between sexes were found in body weight (*p* = 0.002), height (*p* < 0.001), weekly MVPA (*p* < 0.001), and subjective vitality (*p* = 0.012). The sample was recruited through convenience sampling from the participating schools. Table 1 summarizes the anthropometric and sociodemographic characteristics of the participants. No missing observations were present for the variables included in the analyses, and no participants were excluded because of incomplete data. Consequently, no missing-data imputation procedure was required.

### 2.2. Measures

**Dependent variables: Subjective vitality and mental toughness**

Subjective vitality was measured using the Spanish adaptation of the Subjective Vitality Scale validated by Castillo et al. (2017). This tool assesses individuals’ perceived sense of energy, aliveness, and vitality (e.g., “I feel alive and vital”). It consists of seven items answered on a seven-point Likert scale ranging from 1 (strongly disagree) to 7 (strongly agree). Item 2 (“I do not feel very energetic”) demonstrated inadequate psychometric properties and lowered the scale’s internal consistency; therefore, it was removed from the computation of the overall mean score. Item 2 of the Spanish Subjective Vitality Scale was excluded from the final score because it showed inadequate factor loading in the present sample. Higher scores denote greater subjective vitality. The Spanish version of the Subjective Vitality Scale has previously demonstrated psychometric properties and evidence of validity in Spanish-speaking samples (Castillo et al., 2017). In the present sample, the instrument showed good internal reliability (α = 0.89).

Mental toughness was assessed using the eight-item short version proposed by Gucciardi et al. (2015), grounded in the theoretical framework of Clough et al. (2002). Each statement represents a fundamental dimension of the construct: self-efficacy (belief in one’s capacity to succeed), attentional control (ability to concentrate on relevant cues while ignoring distractions), emotional regulation (capacity to identify and manage emotions effectively), achievement-oriented mindset (drive toward success), contextual awareness (understanding and strategically using environmental information), resilience (ability to deal effectively with daily challenges), optimism (expectation of positive outcomes), and coping with adversity (maintaining performance under difficult circumstances). Participants indicated how frequently they experience or apply these behaviors on a seven-point Likert scale (1 = never; 7 = always). A global score was obtained by averaging the eight items, with higher values reflecting greater mental toughness. No additional item deletion or structural modification was performed for the present sample. Internal consistency in this study was acceptable (α = 0.84).

**Independent variables: Weekly MVPA**

Weekly levels of MVPA were evaluated using the PACE+ Adolescent Physical Activity Measure (Prochaska et al., 2001). The questionnaire asks participants to report the number of days during the previous week and during a usual week on which they engaged in at least 60 min of MVPA. The PACE+ is a brief self-report screening measure and therefore provides an estimate of MVPA frequency rather than an objective assessment of accumulated physical activity. As with other self-reported physical activity measures, responses may be affected by recall difficulties, misunderstanding of intensity or duration, and social desirability. Consequently, the reported MVPA values may contain measurement error, which could influence the magnitude of the associations observed with subjective vitality and mental toughness. The final MVPA score was calculated as the mean of both responses: (Q1 + Q2)/2. In accordance with established guidelines (Matias et al., 2018), participants were classified into two categories based on their weekly activity levels: inactive (≤5 days per week of MVPA) and active (>5 days per week of MVPA). This categorization was adopted to maintain consistency with the methodological approach used in previous research.

**Confounding variables: Age, sex and BMI**

Previous evidence suggests that age, sex, and body mass index (BMI) may act as confounding factors affecting both regular physical activity (Martínez-López et al., 2015) and psychological variables in adolescents (Ramírez-Espejo et al., 2025a). Therefore, these variables were included as covariates in the analyses. Body weight and height were measured using an ASIMED^®^ Type B Class III digital scale (Barcelona, Spain) and a SECA^®^ 214 portable stadiometer (SECA Ltd., Hamburg, Germany). Measurements were obtained with participants wearing light clothing and no footwear. BMI was calculated by dividing body weight (kg) by height squared (m^2^). Sociodemographic information was gathered through a structured questionnaire.

### 2.3. Procedure

The aims and procedures of the study were explained both orally and in written form to the students, as well as to their parents or legal guardians. Permission to conduct the research was obtained from the school administrations and the physical education departments. To protect participants’ privacy, all data were anonymized through coding procedures.

Questionnaires assessing physical activity, subjective vitality, mental toughness, and sociodemographic information were administered in a calm classroom environment. A trained researcher provided standardized instructions and supervised the session, while two research assistants helped minimize distractions and safeguarded confidentiality during both questionnaire completion and anthropometric assessments. The full procedure lasted approximately 30 min.

### 2.4. Statistical Analysis

Descriptive data are expressed as means and standard deviations. Independent-samples Student’s *t*-tests were used to examine sex differences in biometric and continuous variables. Multiple linear regression analyses were conducted to determine the association between weekly MVPA and levels of subjective vitality and mental toughness. Additionally, an analysis of covariance (ANCOVA) was performed to analyze differences in psychological variables between active (>5 days/week MVPA) and inactive (≤5 days/week MVPA) groups. The statistical procedures were applied according to the requirements of the corresponding parametric analyses, with age, sex, and BMI included as covariates in the regression and ANCOVA models. Effect sizes were calculated using Hedges’ g, interpreted as small (0.20), medium (0.50), or large (0.80) (Martínez-López et al., 2018). Furthermore, the relative difference between groups was estimated using the formula: [(larger value − smaller value)/smaller value] × 100. Observed power (1 − β) for the ANCOVA effects was calculated using the noncentral F distribution with α = 0.05, based on the corresponding observed F statistic and the numerator and denominator degrees of freedom of each ANCOVA model. In all analyses, subjective vitality and mental toughness served as dependent variables, weekly MVPA as the independent variable, and age, sex, and BMI as covariates. The significance level was set at *p* < 0.05. Statistical analyses were performed using SPSS software (v.25.0, IBM Corp., Armonk, NY, USA).

## 3. Results

### 3.1. Multiple Linear Regression Analysis: Weekly MVPA and Subjective Vitality

Table 2 displays the associations between weekly MVPA and subjective vitality, adjusting for age, sex, and BMI. Results indicated that higher levels of weekly MVPA were significantly associated with greater subjective vitality (β = 0.093; standard error (SE) = 0.044; *p* = 0.034), after controlling for the covariates included in the model.

### 3.2. Multiple Linear Regression Analysis Between Weekly MVPA and Mental Toughness

Table 3 presents the associations between weekly MVPA and mental toughness, adjusting for age, sex, and BMI. Higher levels of weekly MVPA were significantly associated with greater mental toughness (β = 0.122; SE = 0.034; *p* < 0.001), independent of age, sex and BMI.

### 3.3. Differences in Subjective Vitality Between Active and Inactive Adolescents (ANCOVA)

**Figure 1:**
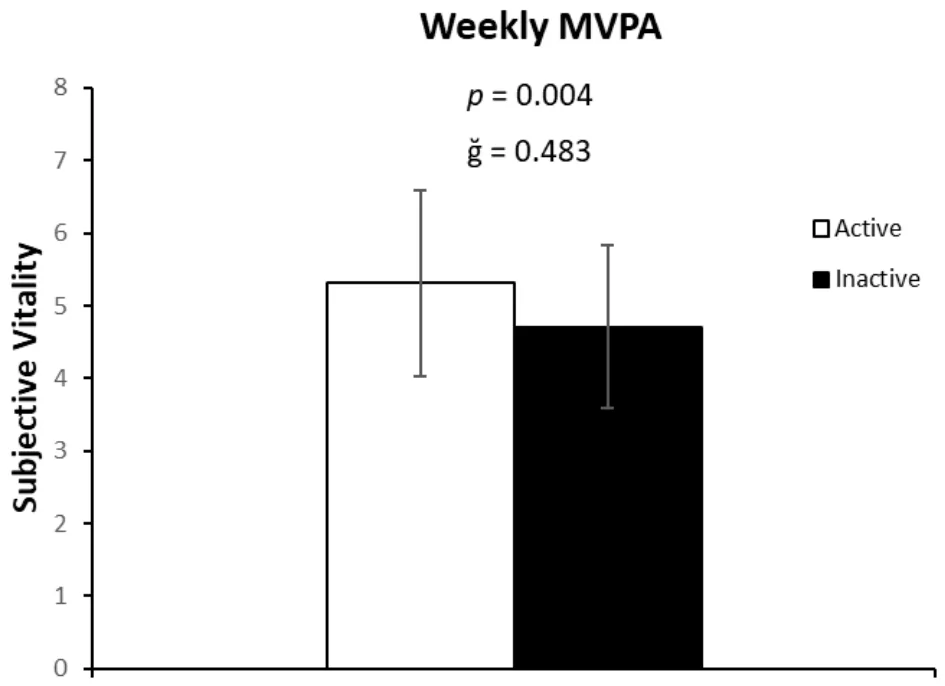
Adjusted mean differences in subjective vitality between higher-frequency MVPA (>5 days/week) and lower-frequency MVPA (≤5 days/week) groups. Means were adjusted for age, sex, and BMI using ANCOVA.

The ANCOVA results comparing subjective vitality levels between active (>5 days per week accumulating at least 60 min of MVPA) and inactive (≤5 days per week with at least 60 min of MVPA) adolescents are illustrated in Figure 1. Active participants showed significantly higher levels of subjective vitality compared to their inactive peers, with a relative difference of 12.7% (5.31 ± 1.12 vs. 4.71 ± 1.28 au), F(1, 277) = 8.328, *p* = 0.004, Hedges’ g = 0.483, 1 − β = 0.820.

### 3.4. Differences in Mental Toughness Between Active and Inactive Adolescents (ANCOVA)

**Figure 2:**
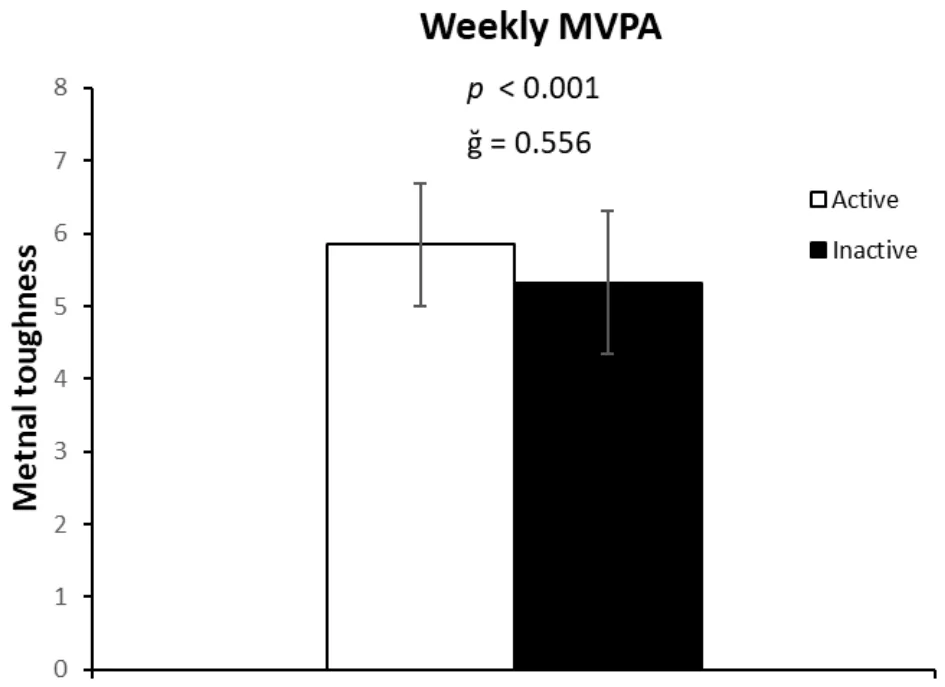
Adjusted mean differences in mental toughness between higher-frequency MVPA (>5 days/week) and lower-frequency MVPA (≤5 days/week) groups. Means were adjusted for age, sex, and BMI using ANCOVA.

The ANCOVA results comparing mental toughness levels between active (>5 days per week accumulating at least 60 min of MVPA) and inactive (≤5 days per week with at least 60 min of MVPA) adolescents are presented in Figure 2. Active participants showed significantly higher levels of mental toughness compared to their inactive peers, with a relative difference of 10.0% (5.85 ± 0.84 vs. 5.32 ± 0.99 au), F(1, 277) = 12.899, *p* < 0.001, Hedges’ g = 0.556, 1 − β = 0.947.

## 4. Discussion

The aim of this study was to analyze the association between weekly MVPA and subjective vitality and mental toughness in Spanish students aged 10 to 16 years, independently of sex, age, and BMI. The main findings showed that higher levels of weekly MVPA were significantly associated with greater subjective vitality and greater mental toughness, regardless of sex, age, and BMI after adjusting for the covariates. In addition, the ANCOVA analyses revealed that active adolescents (>5 days per week with at least 60 min of MVPA) displayed higher levels of both subjective vitality and mental toughness compared to their inactive peers (≤5 days per week), with relative differences of 12.7% and 10.0%, respectively. These results indicate that regular MVPA is consistently related to subjective vitality and mental toughness during adolescence. The findings were consistent with our hypotheses, as higher weekly MVPA was positively associated with both subjective vitality and mental toughness, and the higher-frequency MVPA group showed higher adjusted levels of both psychological outcomes.

The specific contribution of the present study lies in the simultaneous examination of weekly MVPA in relation to two distinct psychological resources, subjective vitality and mental toughness, in Spanish adolescents aged 10–16 years. In addition to examining MVPA as a continuous weekly-frequency measure, the study compared psychological outcomes between higher- and lower-frequency MVPA groups. This complementary analytical approach provides a more differentiated description of the cross-sectional relationship between MVPA frequency and these psychological outcomes than would be obtained from either analysis alone. The data obtained in this study indicate a positive association between weekly MVPA engagement and subjective vitality. Because of the cross-sectional design, this association should not be interpreted as evidence that MVPA directly increases subjective vitality. Such a trend reinforces the consensus that MVPA intensity is consistently associated with higher energy, dynamism, and emotional health within younger cohorts (Kukić et al., 2023). From a motivational standpoint, subjective vitality is deeply rooted in experiences of enjoyment, autonomy, and competence. These psychological nutrients may be supported during activities that demand sustained effort and voluntary involvement (Ryan & Deci, 2008). Furthermore, contemporary research highlights that adolescents integrated into active school settings or those with greater movement opportunities demonstrate a more robust link between physical participation and daily well-being (K. Zhang et al., 2025). Additionally, our analysis showed that physically active adolescents surpassed their inactive peers in terms of vitality levels. This gap in activity profiles aligns with earlier evidence suggesting that active youth not only enjoy better psychological health but also perceive significantly more energy and enthusiasm in their daily routines (Yang et al., 2024). Conversely, lower MVPA levels may limit adolescents’ exposure to physical and psychological stimulation associated with dynamism and perceived competence typically fostered by regular PA (Ruiz-Ranz & Asín-Izquierdo, 2024). Recent findings emphasize that insufficient movement limits exposure to environments that encourage affective activation and intrinsic motivation, both of which are potentially relevant factors associated with subjective vitality (Fu et al., 2025; Ruiz-Ranz & Asín-Izquierdo, 2024).

Our results also indicated a positive cross-sectional association between weekly MVPA and mental toughness. This positive relationship is consistent with literature suggesting that regular participation in MVPA is associated with higher levels of psychological resources related to self-confidence, the ability to face challenges, and perseverance in the presence of adversity (Ramer et al., 2021; Stamp et al., 2017; G. Zhang et al., 2024). Several studies suggest that regular engagement in physical activities involving effort, progression, and a certain degree of challenge may be associated with stronger psychological resources related to mental toughness (Cowden, 2017; Madigan & Nicholls, 2017). Our findings align with this evidence, showing that MVPA provides frequent opportunities to cultivate resilience, emotional control, and frustration tolerance. Likewise, physically active adolescents displayed higher levels of mental toughness compared to their less active peers. This difference is consistent with studies reporting that active youth tend to possess a greater capacity to manage pressure, higher perseverance, and a more adaptive coping style than those who engage in little or no PA (G. Zhang et al., 2024; Madigan & Nicholls, 2017). In contrast, adolescents with insufficient levels of MVPA are typically less exposed to demanding situations requiring effort, self-regulation, and the progressive overcoming of obstacles—conditions that may be relevant to these psychological qualities (Li et al., 2024).

The positive relationship observed between MVPA, subjective vitality, and mental toughness can be interpreted through a biopsychosocial perspective that integrates physiological, psychological, and social processes. At the biological level, MVPA contributes to regulating the hypothalamic–pituitary–adrenal axis, mitigating cortisol secretion while promoting the release of neurotransmitters such as serotonin and dopa-mine, which are closely linked to mood, motivation, and energy (Cortés-Cortés et al., 2019). These neuroendocrine adaptations create a physiological environment conducive to emotional stability, stress management, and the perception of vitality (Belcher et al., 2021). From a psychological perspective, participating in physical activities that involve achievable challenges and progression is associated with higher perceived competence, self-esteem, and self-confidence, all of which are factors associated with greater well-being and mental toughness (Brusseau et al., 2018; Csabai et al., 2024). In structured or group-based practice settings, experiences of cooperation, social support, and belonging synergistically contribute to this effect, strengthening both vitality and resilience under pressure (Sanz-Martín, 2020). Finally, at the contextual and social level, MVPA often takes place in environments offering immediate feedback, meaningful interaction, and shared challenges, conditions that may support perseverance, frustration tolerance, and emotional regulation (Wang et al., 2025). Importantly, the directionality of these associations cannot be established from the present cross-sectional data. Reverse causality is also plausible; adolescents who experience greater subjective vitality or possess higher mental toughness may be more likely to initiate, maintain, or report regular MVPA. Therefore, the observed relationships may reflect bidirectional processes or the influence of unmeasured common determinants rather than an effect of MVPA on psychological functioning.

### 4.1. Practical Recommendations

The findings of this study indicate that higher levels of MVPA are associated with higher subjective vitality and mental toughness in adolescents. Based on these results, we advocate for the promotion of regular MVPA participation, adhering to the guideline of accumulating at least 60 min a day for more than five days a week. To support potential psychological benefits, it is advisable to combine structured activities (e.g., school sports, training programs, or instructor-led sessions) with unstructured, autonomous options such as active play, cycling, dancing, or outdoor recreation. This combination of structure and autonomy may provide a favorable context associated with energy, enjoyment, self-efficacy, and resilience. However, this interpretation should be tested using longitudinal or experimental designs.

From a family perspective, it is essential to consolidate active lifestyle habits by providing emotional support for MVPA participation. This entails facilitating access to sports activities, curbing sedentary screen time, and encouraging daily routines that incorporate movement. Furthermore, family environments that prioritize effort, perseverance, and enjoyment over competitive outcomes contribute to the development of self-confidence and the ability to confront challenges, which are factors potentially relevant to mental toughness. In the educational context, schools should extend their focus beyond Physical Education classes to integrate movement opportunities throughout the entire school day. Initiatives such as active breaks, organized recess activities, sports clubs, or extracurricular programs can significantly increase students’ exposure to MVPA. Physical Education teachers occupy an important role in this process by implementing cooperative methodologies, providing motivational feedback, and setting personal goals that bolster students’ competence and autonomy. Moreover, it would be beneficial to incorporate curriculum content addressing mental health, emotional regulation, and the psychological benefits of PA, thereby enabling students to better understand the potential psychological correlates of physical activity participation. Finally, at the community and policy level, efforts to ensure access to safe and adequate sports infrastructure may be warranted, particularly for vulnerable populations. The implementation of municipal programs, sports scholarships, and well-equipped public spaces can mitigate economic and social barriers that hinder MVPA participation. Likewise, awareness campaigns targeting families, educators, and policymakers could enhance social understanding of the importance of MVPA for adolescents’ psychological well-being, reinforcing the value of physical activity not merely for physical health, but as a behavioral factor potentially associated with emotional vitality and psychological resources such as mental toughness.

### 4.2. Strengths, Limitations and Future Directions

This study presents several limitations that warrant consideration when interpreting the results. First, the use of convenience sampling and the recruitment of participants from schools within a single region of southern Spain limit the generalizability of the findings to other geographical and sociocultural contexts. Second, the cross-sectional design precludes the establishment of causal relationships between MVPA and the psychological variables, making it impossible to determine the precise directionality of the observed associations. Third, MVPA was assessed exclusively through self-report using the PACE+ measure. Although this instrument is practical for school-based screening, self-reported activity may be subject to recall and social desirability biases and does not provide objective estimates of daily activity duration or intensity. The absence of objective measures, such as accelerometry, limits the precision with which MVPA exposure can be characterized and may have affected the strength of the observed associations. Future studies should combine validated self-report measures with objective monitoring to improve the accuracy of MVPA assessment. Another significant limitation is the absence of potentially influential psychosocial factors, such as the specific type of PA (individual vs. team-based), autonomous motivation, family support, or the school’s motivational climate, which could mediate or moderate the relationship between MVPA, subjective vitality, and mental toughness.

Despite these limitations, the study possesses notable strengths. Primarily, it specifically examines the association of weekly MVPA with subjective vitality and mental toughness in Spanish adolescents, a demographic that remains underrepresented in this specific area of scientific literature. Additionally, the inclusion of a substantial and heterogeneous sample of students aged 10 to 16 years offers valuable insights into the educational context analyzed. Furthermore, the statistical control of confounding variables such as sex, age, and BMI strengthens the internal validity of the findings; however, residual confounding cannot be excluded because several potentially relevant variables were not assessed. Lastly, the utilization of psychometrically robust instruments to assess subjective vitality and mental toughness ensures methodological rigor and facilitates comparison with prior research.

Future research should prioritize longitudinal or experimental designs to elucidate the temporal evolution of these associations and establish more robust causal inferences. It is also advisable to incorporate objective PA measurements, such as accelerometry or heart rate monitoring, to obtain a more precise assessment of MVPA levels. Similarly, future studies should analyze mediating and moderating variables to deepen the understanding of the biopsychosocial mechanisms underlying these relationships. Finally, exploring which specific components of MVPA (e.g., intensity, duration, task diversity, or mode of practice) are most effective for optimizing subjective vitality and mental toughness would be of great value. Addressing these dimensions will enable the development of more tailored and effective interventions within educational and community settings.

## 5. Conclusions

This study provides evidence that weekly MVPA is positively associated with higher levels of subjective vitality and mental toughness in adolescents aged 10 to 16 years, irrespective of sex, age, and BMI. The analyses revealed that higher weekly MVPA levels correspond to increases in perceived energy, psychological dynamism, and adaptive resources for coping with pressure and challenges. Furthermore, physically active adolescents (>5 days/week with at least 60 min of MVPA) exhibited significantly superior levels of subjective vitality (+12.7%) and mental toughness (+10.0%) compared to their inactive peers. Taken together, these findings underscore that MVPA is not only related to physical well-being but is also positively associated with subjective vitality and mental toughness during adolescence. Consequently, promoting accessible, varied, and structured PA opportunities across school, family, and community settings may contribute to supporting adolescents’ emotional regulation, perceived energy, motivation, and resilience in their daily lives. Future research should employ longitudinal designs to elucidate the causal direction of these relationships and examine the influence of mediating variables such as motivation, social support, and the specific sport-related context.

## Figures and Tables

**Table 1:** Sociodemographic characteristics, weekly MVPA, subjective vitality, and mental toughness stratified by sex.

Variables	All n = 286		Boys n = 136		Girls n = 150		
	Mean	SD	Mean	SD	Mean	SD	*p*
**Age (Years)**	13.34	1.61	13.44	1.59	13.25	1.62	0.307
**Weight (kg)**	53.88	13.82	56.48	14.04	51.45	13.20	0.002
**Height (m)**	1.60	0.10	1.63	0.11	1.57	0.08	<0.001
**BMI (kg/m^2^)**	21.02	4.53	21.11	4.08	20.93	4.93	0.739
**Weekly MVPA**	4.07	1.76	4.58	1.69	3.60	1.68	<0.001
**Subjective vitality**	4.86	1.26	5.06	1.26	4.68	1.24	0.012
**Mental toughness**	5.45	0.97	5.57	1.03	5.35	0.91	0.064

Note. All variables are presented as mean and standard deviation. BMI = body mass index; MVPA = moderate-to-vigorous physical activity. SD = standard deviation.

**Table 2:** Association between weekly MVPA and subjective vitality adjusted for age, sex, and BMI.

Variable	Subjective Vitality		
	Β	SE	*p*
**Age (Years)**	−0.142	0.047	0.003
**Sex**	−0.307	0.153	0.046
**BMI (kg/m^2^)**	0.001	0.017	0.965
**Weekly MVPA**	0.093	0.044	0.034

Note. Standardized beta (β); standard error (SE); BMI = body mass index (kg/m^2^); MVPA = moderate-to-vigorous physical activity. Sex was coded as [1 = boy, 2 = girl].

**Table 3:** Association between weekly MVPA and mental toughness adjusted for age, sex, and BMI.

Variable	Mental Toughness		
	β	SE	*p*
**Age (Years)**	−0.070	0.036	0.054
**Sex**	−0.107	0.118	0.367
**BMI (kg/m^2^)**	−0.007	0.013	0.556
**Weekly MVPA**	0.122	0.034	<0.001

Note. Standardized beta (β); standard error (SE); BMI = body mass index (kg/m^2^); MVPA = moderate-to-vigorous physical activity [1 = boy, 2 = girl].

## Data Availability

The datasets generated and analyzed during the current study are available from the corresponding author upon reasonable request. Due to the sensitive nature of the information collected, participants were assured that their data would remain confidential and would not be publicly disclosed.

## Abbreviations

The following abbreviations are used in this manuscript:

MVPA	Moderate-to-vigorous physical activity
BMI	Body Mass Index
PA	Physical Activity
